# Comprehensive Re-Sequencing of Adrenal Aldosterone Producing Lesions Reveal Three Somatic Mutations near the KCNJ5 Potassium Channel Selectivity Filter

**DOI:** 10.1371/journal.pone.0041926

**Published:** 2012-07-27

**Authors:** Tobias Åkerström, Joakim Crona, Alberto Delgado Verdugo, Lee F. Starker, Kenko Cupisti, Holger S. Willenberg, Wolfram T. Knoefel, Wolfgang Saeger, Alfred Feller, Julian Ip, Patsy Soon, Martin Anlauf, Pier F. Alesina, Kurt W. Schmid, Myriam Decaussin, Pierre Levillain, Bo Wängberg, Jean-Louis Peix, Bruce Robinson, Jan Zedenius, Martin Bäckdahl, Stefano Caramuta, K. Alexander Iwen, Johan Botling, Peter Stålberg, Jean-Louis Kraimps, Henning Dralle, Per Hellman, Stan Sidhu, Gunnar Westin, Hendrik Lehnert, Martin K. Walz, Göran Åkerström, Tobias Carling, Murim Choi, Richard P. Lifton, Peyman Björklund

**Affiliations:** 1 Department of Surgical Sciences, Genetics and Pathology, Uppsala University, Uppsala, Sweden; 2 Department of Surgery, School of Medicine, Yale University, New Haven, Connecticut, United States of America; 3 Department of General, Visceral and Pediatric Surgery, University Hospital Düsseldorf, Düsseldorf, Germany; 4 Department of General, Visceral and Vascular Surgery, University Hospital, University of Halle-Wittenberg, Halle/Saale, Germany; 5 Department of Molecular Medicine and Surgery, Endocrine Surgery Unit, Karolinska Institutet, Karolinska University Hospital, Stockholm, Sweden; 6 Center for Molecular Medicine, Karolinska Institutet, Karolinska University Hospital, Stockholm, Sweden; 7 Department of Endocrinology, Diabetes and Rheumatology, University Hospital Düsseldorf, Düsseldorf, Germany; 8 Institute of Pathology, University Hospital Düsseldorf, Düsseldorf, Germany; 9 Department of Immunology, Genetics and Pathology, Uppsala University, Uppsala, Sweden; 10 Department of Genetics, School of Medicine, Yale University, New Haven, Connecticut, United States of America; 11 University of Sydney, Endocrine Surgical Unit and Cancer Genetics, Hormones and Cancer Group , Kolling Institute of Medical Research, Royal North Shore Hospital, Sydney, Australia; 12 Klinik für Chirurgie und Zentrum für Minimal Invasive Chirurgie, Kliniken Essen-Mitte, Universität Duisburg-Essen, Essen, Germany; 13 Institut für Pathologie und Neuropathologie Universitätsklinikum, Universität Duisburg-Essen, Essen, Germany; 14 Department of Pathology, Centre Hospitalier Lyon Sud, Lyon, France; 15 Department of Endocrine Surgery, Centre Hospitalier Lyon Sud, Lyon, France; 16 Department of Pathology, University Hospital Lübeck, Lübeck, Germany; 17 Department of Pathology, Marienhospital, Hamburg, Germany; 18 Medizinischen Klinik Universitätsklinikum Schleswig-Holstein, Campus Lübeck, Lübeck, Germany; 19 Endocrine Surgery, Centre Hospitalier Poitiers, Poitiers, France; 20 Pathology Department, Centre Hospitalier Poitiers, Poitiers, France; 21 Sahlgrenska akademin, Göteborg University, Göteborg, Sweden; 22 Department of Surgery, Bankstown Hospital, South Western Sydney Clinical School, University of New South Wales, Sydney, Australia; The Chinese University of Hong Kong, Hong Kong

## Abstract

**Background:**

Aldosterone producing lesions are a common cause of hypertension, but genetic alterations for tumorigenesis have been unclear. Recently, either of two recurrent somatic missense mutations (G151R or L168R) was found in the potassium channel *KCNJ5* gene in aldosterone producing adenomas. These mutations alter the channel selectivity filter and result in Na^+^ conductance and cell depolarization, stimulating aldosterone production and cell proliferation. Because a similar mutation occurs in a Mendelian form of primary aldosteronism, these mutations appear to be sufficient for cell proliferation and aldosterone production. The prevalence and spectrum of *KCNJ5* mutations in different entities of adrenocortical lesions remain to be defined.

**Materials and Methods:**

The coding region and flanking intronic segments of *KCNJ5* were subjected to Sanger DNA sequencing in 351 aldosterone producing lesions, from patients with primary aldosteronism and 130 other adrenocortical lesions. The specimens had been collected from 10 different worldwide referral centers.

**Results:**

G151R or L168R somatic mutations were identified in 47% of aldosterone producing adenomas, each with similar frequency. A previously unreported somatic mutation near the selectivity filter, E145Q, was observed twice. Somatic G151R or L168R mutations were also found in 40% of aldosterone producing adenomas associated with marked hyperplasia, but not in specimens with merely unilateral hyperplasia. Mutations were absent in 130 non-aldosterone secreting lesions.

*KCNJ5* mutations were overrepresented in aldosterone producing adenomas from female compared to male patients (63 vs. 24%). Males with *KCNJ5* mutations were significantly younger than those without (45 vs. 54, respectively; p<0.005) and their APAs with *KCNJ5* mutations were larger than those without (27.1 mm vs. 17.1 mm; p<0.005).

**Discussion:**

Either of two somatic *KCNJ5* mutations are highly prevalent and specific for aldosterone producing lesions. These findings provide new insight into the pathogenesis of primary aldosteronism.

## Introduction

Primary aldosteronism was first described by Conn in 1955, who subsequently on basis of a collected series of patients predicted a prevalence of ∼10% among patients with essential hypertension [1], [2]. After remaining a rare disease for several decades, more recent screening studies have, as Conns predicted, revealed primary aldosteronism as the most common form of secondary hypertension with prevalence of 10% or more in hypertensive patient populations [2], [3], [4], [5], [6], [7]. A surgically curable subtype has been revealed in half of the primary aldosteronism cases (∼5% of the hypertensive patient population), with an even higher prevalence among patients with severe, therapy resistant hypertension [6], [7], [8]. Primary aldosteronism is characterized by inappropriately high, autonomous aldosterone secretion, associated with low serum renin concentrations. Hypersecretion of aldosterone causes increased renal sodium retention and potassium excretion, and the diagnosis was in the past only recognized in hypertensive patients with hypokalemia [2], [6], [9]. Due to recent efficient screening studies, normokalemic presentation has been encountered in a majority of patients (60%), and hypokalemia has been present only in more severe cases [2], [4], [6], [9]. The interest and efforts of screening detection has increased also due to recent recognition of specific, severe cardiovascular morbidity and mortality associated with the aldosterone excess [2], [6], [9], [10], [11], [12].

Primary aldosteronism is caused by adrenocortical adenomas (APAs) or idiopathic hyperplasia, which can be either uni- or bilateral [9]. Patients with primary aldosteronism are detected by raised plasma aldosterone concentration/renin activity or renin concentration ratio (PAC/PRA/or PRC ratio), together with variably raised plasma aldosterone. The diagnosis is confirmed by failure to suppress aldosterone secretion with salt loading, fludrocortisone or ACE inhibitors [2], [9], [13], [14], [15]. Patients with adenoma or unilateral hyperplasia are successfully treated by laparoscopic surgery after identification of an adrenocortical lesion by radiology (CT, MRI), and lateralization by adrenal vein sampling, whereas idiopathic, or micronodular bilateral hyperplasia is managed medically [2], [6], [9], [13], [14], [15], [16]. The distinction may be difficult due to a continuum of physiological and pathological aberrations including solitary, unilateral adenoma, bilateral adenoma, unilateral hyperplasia, bilateral micronodular or macronodular hyperplasia, and adenoma together with non-functioning nodules, known to occur more frequently in elderly individuals [9], [14], [16].

Recently, exome sequencing has identified either of two recurrent somatic mutations (G151R and L168R) in the inwardly rectifying potassium channel *KCNJ5* (*Kir3.4, GIRK4*) in APAs [17], these results were verified in other studies [18], [19], [20], [21]. Germline *KCNJ5* mutations (G151R, G151E and T158A) were also reported to cause a rare dominant form of primary aldosteronism, Familial hyperaldosteronism type III [17], [22], [23]. All these mutations were shown to alter the selectivity filter of KCNJ5, resulting in a channel that can conduct Na^+^ as well as K^+^. Increased Na^+^ conductance results in cell depolarization, which activates voltage-gated Ca^2+^ channels, thereby increasing intracellular Ca^2+^; increased Ca^2+^ is the normal signal for increased aldosterone production and cell proliferation [24]. We now report analysis of *KCNJ5* gene mutations in a large multi-center cohort of adrenocortical tumors.

## Methods

### Ethics Statement

All patients gave written informed consent and approval from these local ethical committees were obtained: Regional ethics committee Uppsala, Regional ethics committee Stockholm, Yale Human Investigation Committee, Local ethics committee at the University of Wuerzburg, University Ethics Committee, University of Halle, Northern Sydney Human Research Ethics Committee, Ethics Committee of the University of Essen, Research and Ethics Committee of the Hospices Civils de Lyon, Local ethics committee of the University of Luebeck, Poitiers Hospital Ethics Committee, Regional ethics committee Gothenburg and South Western Sydney Local Health District Human Research Ethics Committee.

### Patients

Histopathological adrenocortical specimens were collected from 348 patients with clinically diagnosed, apparently sporadic and nonsyndromic, primary aldosteronism, subjected to adrenalectomy at 10 different hospitals, Uppsala and Stockholm, Sweden; Hamburg, Lübeck, Düsseldorf, Essen, and Halle, Germany; Sydney, Australia; Lyon and Poitiers, France. The clinical diagnosis had been established by raised aldosterone/renin ratio together with positive confirmatory tests and lateralisation studies (CT, MRI and adrenal vein sampling) according to the routine protocols at the various centers. The samples were collected from patients with unilaterally dominant lesions based on preoperative lateralization studies, and confirmed by histopathology. The histopathologic diagnosis had been confirmed by expert endocrine pathologists at the different centers. The specimens were categorized into 1) adenoma (without marked associated hyperplasia) 2) adenoma with marked associated hyperplasia, 3) merely hyperplasia of micro-or macronodular type. In addition, three adrenocortical carcinomas with aldosterone excess were included in this study.

130 non aldosterone secreting adrenocortical tumors (Table S2) collected at surgery in Uppsala, were also subjected to study.

### DNA and RNA extraction, RT-PCR and immunohistochemistry

DNA, RNA extraction and subsequent cDNA synthesis were done as previously described [25]. Briefly DNA and RNA were prepared from cryosections using DNeasy Blood & Tissue Kit (Qiagen, Hilden, Germany) or FFPE sections using AllPrep DNA/RNA FFPE Kit (Qiagen, Hilden, Germany). Sections (6 µm) were stained with hematoxylin-eosin to verify presence of tumor cells prior to mutation analysis. From available specimens with clear distinguishable nodules or small adenomas (n = 41), punched needle biopsies (n = 78) were obtained from different areas of the specimens to investigate small adenomas, macronodules, as well as areas with micronodular, or diffuse hyperplasia for mutation analysis. Reverse transcription of RNA was performed with random hexamer primers using the First-Strand cDNA Synthesis kit (GE Healthcare AB, Stockholm, Sweden) according to the manufacturer's instructions. PCR reactions were performed using primers and conditions described in Table S1. RT-PCR of KCNJ5 was performed using mRNA specific primers. 10 ng of RNA (-RT) was used as negative control.

KCNJ5 protein expression was investigated in 64 specimens by immunohisto-chemistry as previously described [17]. The intensity of the staining was scored by two independent observers on a scale of weak, moderate and strong. In addition, the pattern of staining was evaluated as hetero- or homogenous.

### DNA sequencing

All *KCNJ5* coding exons with intron/exon junctions were directly sequenced (Beckman Coulter Genomics, Tackeley, UK) and traces were analyzed using CodonCode Aligner software (CodonCode Corporation, Dedham, MA).

#### Orthologs

Protein sequences were aligned using the ClustalW algorithm. GenBank accession numbers were: NP_000881.3 (human), NP_034735.3 (mouse), XP_417864.2 (chicken), NP_001016901.1 (frog), XP_700619.4 (zebrafish), and XP_002122831.1 (tunicate).

### Statistical analysis

SPSS 18 (IBM, NY, USA) was used for statistical analysis. All results are expressed as mean±SEM and range for non-normally distributed variables. log transformed values were used for analysis of non-symmetric variables. Intra-group changes were analyzed by paired *t* test. ANOVA, mixed model, was used for comparison between two groups based on absence or presence of *KCNJ5* mutations, followed by Chi square test. Mann-Whitney U test was applied for nonparametric analysis. Significant results of univariate tests were analyzed in a multivariate model. A p-value of <0.05 was considered significant.

## Results

### *KCNJ5* mutation status in adrenocortical lesions

**Figure 1:**
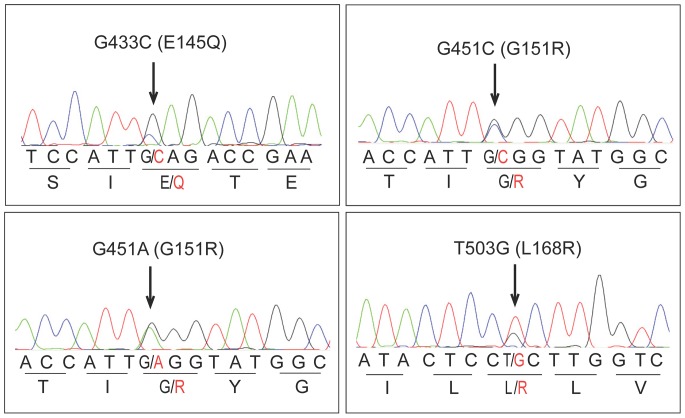
Sanger traces from 4 tumor samples with somatic mutations G433C (pE145Q), G451A and G451C (pG151R) and T503G (pL168R) in *KCNJ5*.

**Figure 2:**
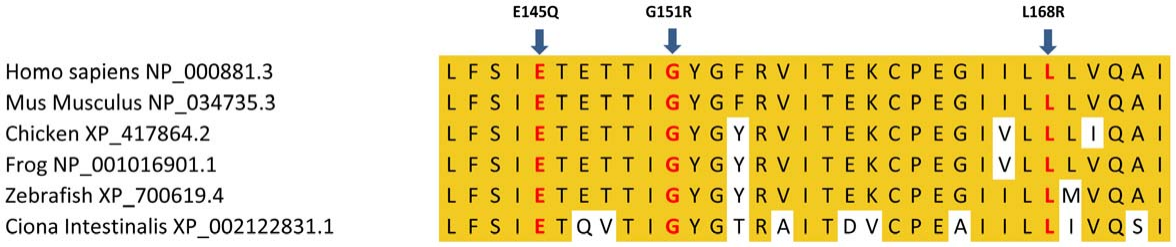
Comparison of different KCNJ5 orthologs. Complete conservation of substituted amino acid residues E145, G151 and L168 across multiple specimens, from Human to Tunicate.

In total *KCNJ5* mutations were identified in 157 of 348 (45%) of aldosterone producing lesions (Table 1). Of these, 155 resulted in the previously reported G151R and L168R substitutions (found in 24% and 20% of all samples, respectively) (Figure 1). In addition, two APAs had a single base substitution resulting in a novel E145Q mutation located near the selectivity filter at a highly conserved position (Figure 1 and 2). These mutations were mutually exclusive. G151R, L168R, T158A [17] as well as I157S [26] and G151E [27] substitutions have been shown to affect protein structure and disturb selectivity filter specificity, thus it is likely that E145Q would also affect the selectivity filter specificity. The L168R mutation was observed in one of three adrenocortical carcinomas with excess aldosterone production. In all 137 cases in which matched DNA from blood or surrounding normal tissue was available, *KCNJ5* mutations were specific to adenomas, consistent with these representing somatic mutations.

**Table 1:** Lesion characteristics and mutation spectrum.

			*KCNJ5* mutations			
Variable	Total cohort	Wild Type	All mutations	G151R	L168R	E145Q
Adenoma without associated hyperplasia - no. (%)	287	151 (53%)	136 (47%)	74 (26%)	60 (21%)	2 (0.7%)
Males - no. (%)	109 (38%)	85 (78%)	24 (22%)†	13 (12%)	11 (10%)	0 (0%)
Females - no. (%)	178 (62%)	66 (37%)	112 (63%)†	61 (34%)	49 (28%)	2 (1.1%)
Age at operation - yr (range)	49 (16–79)	52 (26–79)	46 (16–78)	46 (16–78)	44 (23–72)	46 (45–47)
Males	53 (16–79)	54 (30–79)‡	45 (16–67)‡	46 (16–59)	45 (29–67)	–
Females	47 (23–79)	49 (26–79)	46 (23–78)	47 (26–78)	44 (23–72)	46 (45–47)
Adenoma size - mm (range)	17.3 (6–47)	15.9 (6–47)	18.6 (6–47)	18.9 (6–47)	18.4 (6–40)	8 (6–10)
Males	19.7 (6–47)	17.1 (6–47)¶	27.1 (6–47)¶	32.5 (6–47)¶	18.6 (6–30)	–
Females	18.1 (6–45)	14.9 (6–45)	18.7 (7–40)	19.3 (9–36)	18.3 (7–40)	8 (6–10)
Adenoma with associated hyperplasia - no. (%)	52	31 (60%)	21 (40%)	10 (19%)	11 (21%)	0 (0%)
Males	36 (69%)	25 (69%)	11 (31%)#	4 (11%)	7 (19%)	0 (0%)
Females	16 (31%)	6 (38%)	10 (63%)#	6 (38%)	4 (25%)	0 (0%)
Age at operation - yr (range)	53 (22–73)	54 (40–68)	49 (22–73)	56 (47–73)	42 (22–69)	–
Males	52 (22–68)	56 (40–68)	43 (22–60)	51 (47–60)	39 (22–57)*	–
Females	54 (37–73)	52 (44–65)	55 (37–73)	60 (54–73)	47 (37–69)	–
Hyperplasia - no. (%)	9	9 (100%)	0 (0%)	–	–	–
Males	6 (67%)	6 (67%)	–	–	–	–
Females	3 (33%)	3 (33%)	–	–	–	–
Age at operation - yr (range)	51 (38–62)	51 (38–62)	–	–	–	–
Males	49 (38–58)	49 (38–58)	–	–	–	–
Females	54 (44–62)	54 (44–62)	–	–	–	-

*KCNJ5* mutation spectrum and prevalence. Significant overrepresentation of female patients with a lesion harboring *KCNJ5* mutation, both adenomas and adenomas with associated hyperplasia. Males with adenomas harboring *KCNJ5* mutations were significantly younger at the time of surgery and these adenomas were significantly larger than those without mutation.

†, ‡, ¶ and # indicate p-value<0.005.

Stratifying by lesion type, 136 mutations were found in 287 APAs in which surrounding hyperplasia was not found (47%). Twenty-one mutations were identified in 52 adenomas in which surrounding hyperplasia was observed (40%); in 41 specimens punch biopsies of hyperplastic surrounding tissue did not show *KNCJ5* mutations. No *KCNJ5* mutations were found in 9 specimens with hyperplasia without APA, including sampling selected macronodules of variable size from the same lesion (Table 1). *KCNJ5* mutation (L168R) was also found in one of three aldosterone-secreting adrenocortical carcinomas (33%). In contrast, no *KCNJ5* mutations were detected in 130 non-aldosterone secreting adrenocortical specimens (Table S2). There were no significant differences between different contributing centers (Table S3).

### Gender dimorphism in *KCNJ5* mutation frequency

**Figure 3:**
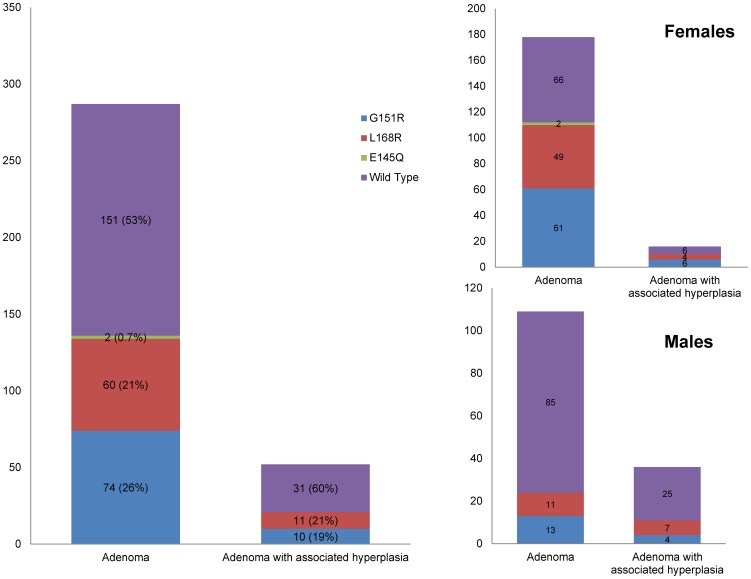
Mutation spectrum and gender distribution. L168R and G151R substitutions account for the waste majority of the mutations found in aldosterone producing lesions. KCNJ5 mutations are more frequent in lesions from female patients, indicating a distinct gender dimorphism.

Genotype-phenotype correlation demonstrated a dramatic difference in the prevalence of *KCNJ5* mutations in women and men (Figure 3). While *KCNJ5* mutations were found in 63% of APA's without surrounding hyperplasia in women (112/178), they were present in only 22% of APA's in males (24/109) (Table 1). This difference, a 2.9∶1 risk ratio in female versus male patients, is statistically significant (p = 10^−11^). A similar female bias for *KCNJ5* mutations was seen among APAs with surrounding hyperplasia (ratio 2.0∶1).

Females with and without *KCNJ5* mutations had surgery at similar ages and their adenomas were of similar size at surgery (Table 1). Males with *KCNJ5* mutations were in average 9 years younger at the time of surgery than those without (45 vs. 54, respectively; p<0.005). APAs with *KCNJ5* mutations in male patients were on average 1 cm larger than those without (27.1 mm vs. 17.1 mm; p<0.005); this was attributable to males with G151R mutations having the largest APAs.

### KCNJ5 expression analysis

**Figure 4:**
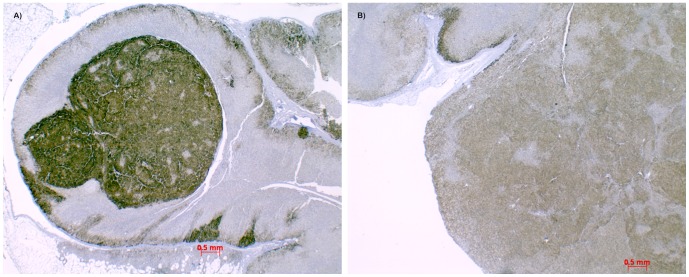
Heterogeneous immunoreactivity of KCNJ5 independent of mutational status. **A**) Intense KCNJ5 reactivity in a 4 mm macronodule expressing only WT *KCNJ5*. **B**) Moderate KCNJ5 reactivity in a 19 mm large adenoma expressing L168R *KCNJ5*.

All lesions examined expressed the mutated allele at the mRNA level as demonstrated by reverse transcriptase PCR using mRNA-specific primers as described in Table S1. KCNJ5 staining using specific antibodies were variable in both adenomas and adenoma-like macronodules, showing intense, weak or heterogenous staining. This was not correlated to *KCNJ5* mutation status (Figure 4).

## Discussion

The present findings confirm and extend the recent discovery of recurrent mutations in *KCNJ5* as a prevalent cause of APA (Table 2). The previously identified mutations resulting in G151R and L168R were found in similar frequencies that together comprise 46% of APAs, which is comparable to other published studies [17], [18], [20]. In addition we found two instances of a previously unidentified mutation, E145Q. Like the others, this mutation lies near the selectivity filter in a highly conserved region and we infer it is also likely to increase Na+ conductance. The increase in Na+ conductance may explain why a number of patients show an increase in PAC despite volume load and suppression of renin in saline infusion tests. In addition, one *KCNJ5* mutation was found in an aldosterone-secreting adrenocortical carcinoma. These findings demonstrate that the G151R and L168R mutations account for nearly 99% of *KCNJ5* mutations in APAs and suggest that few additional mutations in this gene are unlikely to account for significant fractions of APA.

**Table 2:** Summary of reported *KCNJ5* somatic mutations in Aldosterone producing adenomas.

	Sequenced specimens	Mutation frequency (no.)	G151R	L168R	Other mutations
Choi et al. [17]	22	36% (n = 8)	2	6	
Azizan et al. [20]	73	41% (n = 30)	19	10	del I157 (n = 1)
Taguchi et al. [19]	23	65% (n = 15)	12	3	
Boulkroun et al. [18]	380	34% (n = 129)	76	53	
Present study	348	45% (n = 157)	84	71	E145Q (n = 2)

*KCNJ5* mutations were prevalent in tumors in which there was a solitary or dominant nodule; the tumors with mutation were of variable size (6–47 mm) at the time of surgery and patients with and without mutations generally had surgery at similar ages. However male patients with APAs presented with larger tumors and were significantly younger at the time of surgery. This was true in both the presence and absence of surrounding hyperplasia. In contrast, no *KCNJ5* mutations were found among 9 cases prospectively classified as unilateral hyperplasia with or without multiple nodules despite analyzing DNA from most of the available nodules in each sample (p<0.005 for difference in frequency compared with all APAs). Similarly, no *KCNJ5* mutations were found in other non-aldosterone secreting adrenal lesions, demonstrating their specificity for aldosteronism. These observations support a distinct pathophysiology of APA with hyperplasia and unilateral hyperplasia without a dominant nodule.

A striking gender dimorphism in the prevalence of *KCNJ5* mutations were observed in this study and by others [18]. APAs have consistently been found to be more prevalent in women than men with a ratio of about 2∶1 [28]. It appears that this entire excess can be accounted for by the increased prevalence of *KCNJ5* mutations among APAs in women compared to men since we observed a 2.6 – fold increase in the prevalence of *KCNJ5* mutations in female compared to male APAs. APAs without *KCNJ5* mutations actually had a higher prevalence in males than females in our cohort. Whether this gender bias for *KCNJ5* mutation is attributable to a difference in the rate at which these mutations occur in females vs. males or to differences in the likelihood of tumor development following mutation will be of interest to determine.

### Perspectives

The identification of a very small number of mutations that account for a large fraction of APAs indicates that these mutations account for a large number of patients with severe hypertension worldwide. This raises the question of whether specific diagnostic and/or therapeutic approaches may be fruitful. The potential to detect specific somatic mutations in DNA shed into plasma with high sensitivity suggests a potential screening test that could detect a large fraction of APAs noninvasively. Likewise, obtaining a puncture for histology may prove being useful in lateralization of the lesion. Similarly, the specific *KCNJ5* mutations likely result in specific alterations in channel structure that might allow selective inhibition of mutant channels, which would be expected to inhibit aldosterone secretion and arrest progression of tumor growth in affected patients.

## Supporting Information

Table S1 Primers sequences used in PCR and RT-PCR reactions.
(DOCX)

Table S2 Non aldosterone producing lesions characteristics. No *KCNJ5* mutations have been found.
(DOCX)

Table S3 Mutation status in adenomas without associated hyperplasia, shown for each participating center.
(DOCX)
