# Supplementary material for: Comprehensive Re-Sequencing of Adrenal Aldosterone Producing Lesions Reveal Three Somatic Mutations near the KCNJ5 Potassium Channel Selectivity Filter
Source: PLoS One. 2012 Jul 27;7(7):e41926. doi: 10.1371/journal.pone.0041926 (PMC3407065; doi:10.1371/journal.pone.0041926)
Supplement: Table S1 — Primers sequences used in PCR and RT-PCR reactions. (DOCX) [file pone.0041926.s001.docx]

| Fragment 1 | intron 1-2 | CTAGTGAATCAGAACAGCCCAC |
| --- | --- | --- |
|  | exon 2 | AAGGAATCCACTCTTGGTCG |
| Fragment 2 | exon 2 | GCTTCATTTGGTGGCTCATT |
|  | exon 2 | CCACCATGAAGGCATTGAC |
| Fragment 3 | exon 2 | GTGTCCGCTTTCCTGTTCTC |
|  | intron 2-3 | CTAAGTCTGAAGTGTAGGTAG |
| Fragment 4 | intron 2-3 | AATGGATGGATAGATGGATGG |
|  | exon 3 | GTCTGTGTTCACTGAAGCCAG |
| Verification | exon 2 | CGACCAAGAGTGGATTCCTT |
|  | exon 2 | AGGGTCTCCGCTCTCTTCTT |
| cDNA | exon 2 | GCTTCATTTGGTGGCTCATT |
|  | exon 3 | GTCTGTGTTCACTGAAGCCAG |

**Table S1**
