# Supplementary material for: Comprehensive Re-Sequencing of Adrenal Aldosterone Producing Lesions Reveal Three Somatic Mutations near the KCNJ5 Potassium Channel Selectivity Filter
Source: PLoS One. 2012 Jul 27;7(7):e41926. doi: 10.1371/journal.pone.0041926 (PMC3407065; doi:10.1371/journal.pone.0041926)
Supplement: Table S2 — Non aldosterone producing lesions characteristics. No KCNJ5 mutations have been found. (DOCX) [file pone.0041926.s002.docx]

| Table S2 | | | | |  |  |
| --- | --- | --- | --- | --- | --- | --- |
|  |  |  |  |  |  |  |
| **Lesion characteristics** | | |  | **Age at operation - yr (range)** | **Male to female ratio** | **Tumor size** |
|  |  |  |  |  |  |  |
| Cortisol producing adenomas (n=30) | | | | 34 (22-62) | 1:2 | 34 (10-50) |
|  |  | | |  |  |  |
| Cortisol producing carcinomas (n=20) | | | | 38 (28-42) | 1:1.5 | 58 (35-80) |
|  |  |  |  |  |  |  |
| Non functioning adenomas (n=50) | | | | 53 (38-71) | 1:1 | 63 (55-85) |
|  |  | | |  |  |  |
| Non functioning carcinomas (n=30) | | | | 47 (31-74) | 1:1 | 80 (55-210) |
|  |  |  |  |  |  |  |
