# Supplementary material for: Comprehensive Re-Sequencing of Adrenal Aldosterone Producing Lesions Reveal Three Somatic Mutations near the KCNJ5 Potassium Channel Selectivity Filter
Source: PLoS One. 2012 Jul 27;7(7):e41926. doi: 10.1371/journal.pone.0041926 (PMC3407065; doi:10.1371/journal.pone.0041926)
Supplement: Table S3 — Mutation status in adenomas without associated hyperplasia, shown for each participating center. (DOCX) [file pone.0041926.s003.docx]

| Table S3 |  |  |  |  |  |  |
| --- | --- | --- | --- | --- | --- | --- |
|  |  |  |  |  |  |  |
| **Center** | **Number** | **Wild Type** | **All mutations** | **G151R** | **L168R** | **E145Q** |
|  |  |  |  |  |  |  |
| France, Petirie | 19 | 10 | 9 | 4 | 4 | 1 |
|  |  |  |  |  |  |  |
| France, Lyon | 38 | 18 | 20 | 10 | 10 | 0 |
|  |  |  |  |  |  |  |
| Germany, Essen | 52 | 30 | 22 | 14 | 7 | 1 |
|  |  |  |  |  |  |  |
| Germany, Halle | 8 | 4 | 4 | 1 | 3 | 0 |
|  |  |  |  |  |  |  |
| Germany, Düsseldorf | 34 | 20 | 14 | 8 | 6 | 0 |
|  |  |  |  |  |  |  |
| Australia, Sydney | 33 | 18 | 15 | 9 | 6 | 0 |
|  |  |  |  |  |  |  |
| Sweden, Stockholm | 35 | 15 | 20 | 8 | 12 | 0 |
|  |  |  |  |  |  |  |
| Germany, Lübeck | 36 | 16 | 20 | 11 | 9 | 0 |
|  |  |  |  |  |  |  |
| Sweden, Uppsala | 32 | 20 | 12 | 9 | 3 | 0 |
